# Left occipitotemporal cortex contributes to the discrimination of tool-associated hand actions: fMRI and TMS evidence

**DOI:** 10.3389/fnhum.2014.00591

**Published:** 2014-08-05

**Authors:** Francesca Perini, Alfonso Caramazza, Marius V. Peelen

**Affiliations:** ^1^Center for Mind/Brain Sciences, CIMeC, University of TrentoRovereto, Trento, Italy; ^2^Department of Psychology, Harvard UniversityCambridge, MA, USA

**Keywords:** action knowledge, middle temporal gyrus, tool selectivity, hand selectivity, lateral occipitotemporal cortex

## Abstract

Functional neuroimaging studies have implicated the left lateral occipitotemporal cortex (LOTC) in both tool and hand perception but the functional role of this region is not fully known. Here, by using a task manipulation, we tested whether tool-/hand-selective LOTC contributes to the discrimination of tool-associated hand actions. Participants viewed briefly presented pictures of kitchen and garage tools while they performed one of two tasks: in the action task, they judged whether the tool is associated with a hand rotation action (e.g., screwdriver) or a hand squeeze action (e.g., garlic press), while in the location task they judged whether the tool is typically found in the kitchen (e.g., garlic press) or in the garage (e.g., screwdriver). Both tasks were performed on the same stimulus set and were matched for difficulty. Contrasting fMRI responses between these tasks showed stronger activity during the action task than the location task in both tool- and hand-selective LOTC regions, which closely overlapped. No differences were found in nearby object- and motion-selective control regions. Importantly, these findings were confirmed by a TMS study, which showed that effective TMS over the tool-/hand-selective LOTC region significantly slowed responses for tool action discriminations relative to tool location discriminations, with no such difference during sham TMS. We conclude that left LOTC contributes to the discrimination of tool-associated hand actions.

## Introduction

Tools physically and functionally extend our body, allowing us to achieve a wide range of goals that would not be possible with our bodies alone. Much progress has recently been made in understanding the neural architecture that supports complex tool use. Evidence from multiple methods points to a left lateralized network of frontal, parietal, and occipitotemporal brain regions involved in tool use and tool perception (for reviews, see Johnson-Frey, 2004; Lewis, 2006; Martin, 2007; Vingerhoets, 2014). While the involvement of frontal and parietal cortices in tool action processing has been relatively well established, the contribution of the occipitotemporal cortex is still poorly understood. In the present study we used functional magnetic resonance imaging (fMRI) and transcranial magnetic stimulation (TMS) to test whether left occipitotemporal cortex contributes to the discrimination of tool-associated hand actions.

fMRI studies have provided evidence that viewing pictures of tools, relative to other object categories such as animals or chairs, activates the left lateral occipitotemporal cortex (LOTC; Chao et al., 1999; Beauchamp et al., 2002; Valyear et al., 2007; Weisberg et al., 2007; Valyear and Culham, 2010; Bracci et al., 2012). Tool-selective activity in LOTC is also observed in congenitally blind individuals (Peelen et al., 2013), suggesting a role for this region in knowledge of tool actions rather than in representing purely visual properties of tools. However, it remains possible that tool-selective activity in LOTC reflects shape differences between tools and other objects (e.g., elongated tool shape; Sakuraba et al., 2012), or the small size of tools relative to typically used control categories (Konkle and Oliva, 2012). These properties are not strictly visual and might thus still account for tool-selective LOTC activity in the congenitally blind. Although patient studies have broadly supported a role for left posterior temporal cortex in conceptual action knowledge (Tranel et al., 2003; Campanella et al., 2010; Kalenine et al., 2010), it is not clear from these studies whether lesions to tool-selective LOTC, more anterior regions in middle temporal gyrus (MTG), or other co-lesioned regions caused these deficits. Moreover, recent fMRI evidence sheds new light on the putative role of left LOTC in tool perception by showing that tool-selective regions closely overlap with hand-selective regions (Bracci et al., 2012). This finding raises the possibility that left LOTC contributes to tool action discrimination by accessing tool-associated hand action or hand posture representations.

In the present fMRI study, we tested whether left LOTC contributes to the discrimination of tool-associated hand actions by comparing fMRI activity while participants performed two different tasks on the same stimulus set. In the action task, they judged whether a tool is associated with a hand rotation action (e.g., screwdriver) or a hand squeeze action (e.g., garlic press), while in the location task they judged whether a tool is typically found in the kitchen (e.g., garlic press) or in the garage (e.g., screwdriver). If tool-selective LOTC is involved in processing action-related properties of tools, we expect an increase of activity when participants pay attention to action properties relative to when they focus on other properties such as a tool's typical location.

Previous fMRI studies adopting a similar task-comparison approach failed to find action-specific activity in LOTC. For example, one fMRI study contrasted action knowledge with function knowledge of tools (Canessa et al., 2008). In the action knowledge task, participants were asked whether two objects (presented simultaneously for 4 s) were used with the same manipulation pattern (e.g., vacuum cleaner and metal detector). Activity for this task was contrasted with a function knowledge task, in which participants had to judge whether the two objects were used in the same functional context (e.g., vacuum cleaner and carpet beater). The contrast between action knowledge and function knowledge of tools yielded significant activation in left dorsal premotor cortex and left IPS, but not in left LOTC. Other imaging studies that contrasted manipulation with function knowledge of manipulable objects similarly found increased activity in parietal cortex but not in LOTC (Kellenbach et al., 2003; Boronat et al., 2005; Bach et al., 2010).

These results might be interpreted as evidence that LOTC activity in response to tools is fully driven by visual, shape, or size properties of tools (common to both action and function tasks). Alternatively, however, it may be that LOTC is activated by both action and function judgments, as both these tasks require access to tool-specific action knowledge: judging whether or not a vacuum cleaner and a carpet beater are used in the same functional context requires knowledge of what these objects are used for. Therefore, in the present study, we contrasted tool action discrimination with tool location discrimination. Discriminating the typical location of a tool does not require access to tool-specific action or function knowledge; indeed, this task can be equally performed on non-tool objects.

Using fMRI, we found a strong and anatomically specific increase of activity in hand- and tool-selective LOTC regions for the action relative to the location task. In a subsequent TMS study, we found that effective TMS (relative to sham TMS) over hand-/tool-selective left LOTC differentially affected performance on tool action as compared to tool location discriminations, such that effective TMS significantly slowed responses on the action task relative to the location task, with no such difference during sham TMS. These results suggest that left LOTC causally contributes to the discrimination of tool-associated hand actions.

## Materials and methods

### fMRI experiment

#### Participants

Fourteen healthy adult volunteers (6 females; mean age: 26.8 years, age range: 20–35 years) participated in the fMRI experiment. One participant was excluded because of low accuracy in the main experiment (> 2 standard deviations below the group mean). All participants were right-handed with normal or corrected-to-normal vision, and no history of neurological or psychiatric disease. Participants gave written informed consent for participation in the study, which was approved by the human research ethics committee of the University of Trento.

#### Stimuli

**Figure 1:**
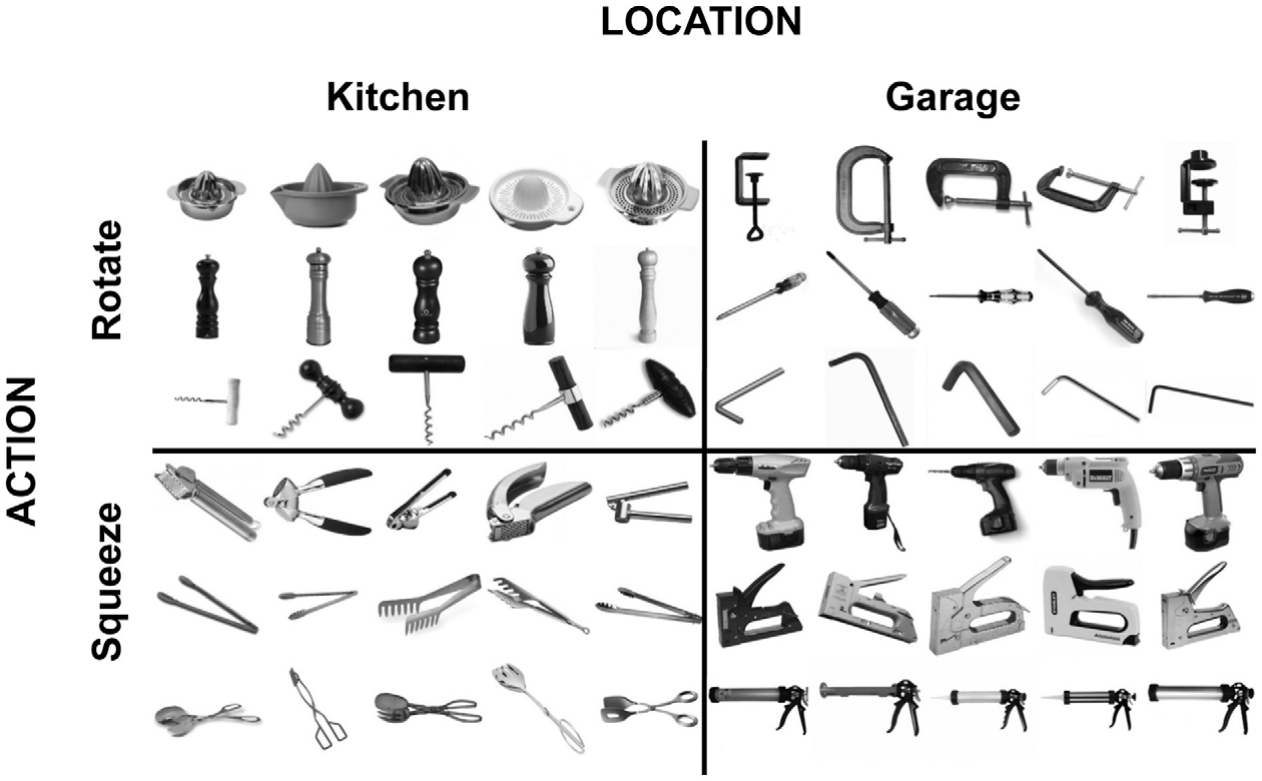
**Stimuli used in the fMRI and TMS experiments**.

The stimulus set (Figure 1) consisted of 5 different exemplars of 12 objects. Half of the objects are typically found in a kitchen, and the other half in a garage. Half of the objects are manipulated by a wrist rotation movement, and the other half by a hand-squeeze movement.

Stimuli (400 × 400 pixels, 5°) were presented centrally. Stimulus presentation was controlled by a PC running the Psychophysics Toolbox package (Brainard, 1997) in Matlab (Mathworks, Natick, MA, USA). Pictures were projected onto a screen and were viewed through a mirror mounted on the head coil.

#### Task and design of main fMRI experiment

Participants performed a 1-back task, detecting repetitions of either the location (kitchen, garage) or the action (rotate, squeeze) dimension of the objects. The tasks were performed in different runs, with the order counterbalanced across participants, such that 7 of the 14 participants started with the action task and 7 of the 14 participants started with the location task. Participants performed a total of 6 runs of 120 trials each. On each trial, a picture of a tool object (Figure 1) was presented centrally for 1200 ms, followed by a 600 ms fixation period. Each of the 12 tool objects was presented 10 times within a run, in random order. The exemplar of the object that was presented (e.g., which of the 5 corkscrews; Figure 1) was randomly selected on each trial. Participants pressed a response button with their right index finger when a task-relevant repetition occurred. Detection performance (1-back repetition detection) was high for both tasks [action task: mean = 97.2% correct; location task: mean = 97.9% correct; difference: *t*_(12)_ = 2.1, *P* = 0.06].

### Functional localizers

Twelve (of the total of 14) participants additionally participated in three functional localizer experiments.

#### Category localizer

The category localizer consisted of two runs lasting 5 min each. The experiment consisted of four conditions: tools, animals, hands, and outdoor scenes. Stimuli (400 × 400 pixels, 12°) were presented centrally and consisted of isolated objects on a white background (see Bracci et al., 2012, for examples). One scanning run consisted of 21 blocks of 14 s each. Blocks 1, 6, 11, 16, and 21 were fixation-only baseline epochs. In each of the remaining blocks, 20 different stimuli from one category were presented. These stimuli were randomly selected from a total set of 40 stimuli per category. Each stimulus appeared for 350 ms, followed by a blank screen for 350 ms. Twice during each block, the same picture was presented two times in succession. Participants were required to detect these repetitions and report them with a button press (1-back task). Each participant was tested with two different versions of the experiment that counterbalanced for the order of the blocks. In both versions, assignment of category to block was counterbalanced, so that the mean serial position in the scan of each condition was equated.

#### Object localizer

Participants performed one run of a standard object-selective cortex localizer, lasting 5 min (Downing et al., 2007; Bracci et al., 2012). Stimuli consisted of 20 intact and 20 scrambled objects, which were presented in alternating blocks. The block structure and task were identical to the category localizer experiment.

#### Motion localizer

To localize motion-selective cortex, visual displays of moving and stationary dot patterns were presented either in the left visual field (LVF) or in the right visual field (RVF). In the motion condition, dots shifted from the starting position toward the display's edge and back toward the fixation (0.5°/s) alternating direction every three frames. In the static condition the dots remained still. The single localizer run lasted 8 min 48 s during which the four stimulus conditions (static dots in the LVF, moving dots in the LVF, static dots in the RVF and moving dots in the RVF), each lasting 16 s, were interleaved with fixation blocks (16 s). Each stimulus condition was repeated four times in a random order within the run. Fixation blocks also appeared at the beginning and end of each run. The fixation point changed color (red, yellow, green, blue) every 500 ms. To maintain attention, participants were instructed to press a button with their right index finger whenever the central fixation point turned red.

#### fMRI data acquisition

Functional and structural MRI data were collected at the Center for Mind/Brain Sciences, University of Trento, Italy. All images were acquired on a Bruker BioSpin MedSpec 4-T scanner (Bruker BioSpin GmbH, Rheinstetten, Germany). Functional images were acquired using echo planar (EPI) T2^*^-weighted scans. Acquisition parameters were: repetition time (TR) of 2 s, an echo time (TE) of 33 ms, a flip angle (FA) of 73°, a field of view (FoV) of 192 mm, and a matrix size of 64 × 64. Each functional acquisition consisted of 34 axial slices (which covered the whole cerebral cortex) with a thickness of 3 mm and gap of 33% (1 mm). Structural images were acquired with an MP-RAGE sequence with 1 × 1 × 1 mm resolution.

#### fMRI data preprocessing

Data were analyzed using the AFNI software package (http://afni.nimh.nih.gov/) and MATLAB (The MathWorks, Natick, MA). Functional data were slice-time corrected, motion corrected, and low-frequency drifts were removed with a temporal high-pass filter (cutoff of 0.006 Hz). All data were spatially smoothed (4 mm Gaussian kernel) and transformed into Talairach space, which included resampling to 3 × 3 × 3 mm voxels.

#### fMRI data analysis

For each participant, general linear models were created to model the conditions in the experiment. All trials were included in the analyses. Regressors of no interest were also included to account for differences in the mean MR signal across scans and for head motion within scans.

#### Region of interest definition

**Figure 2:**
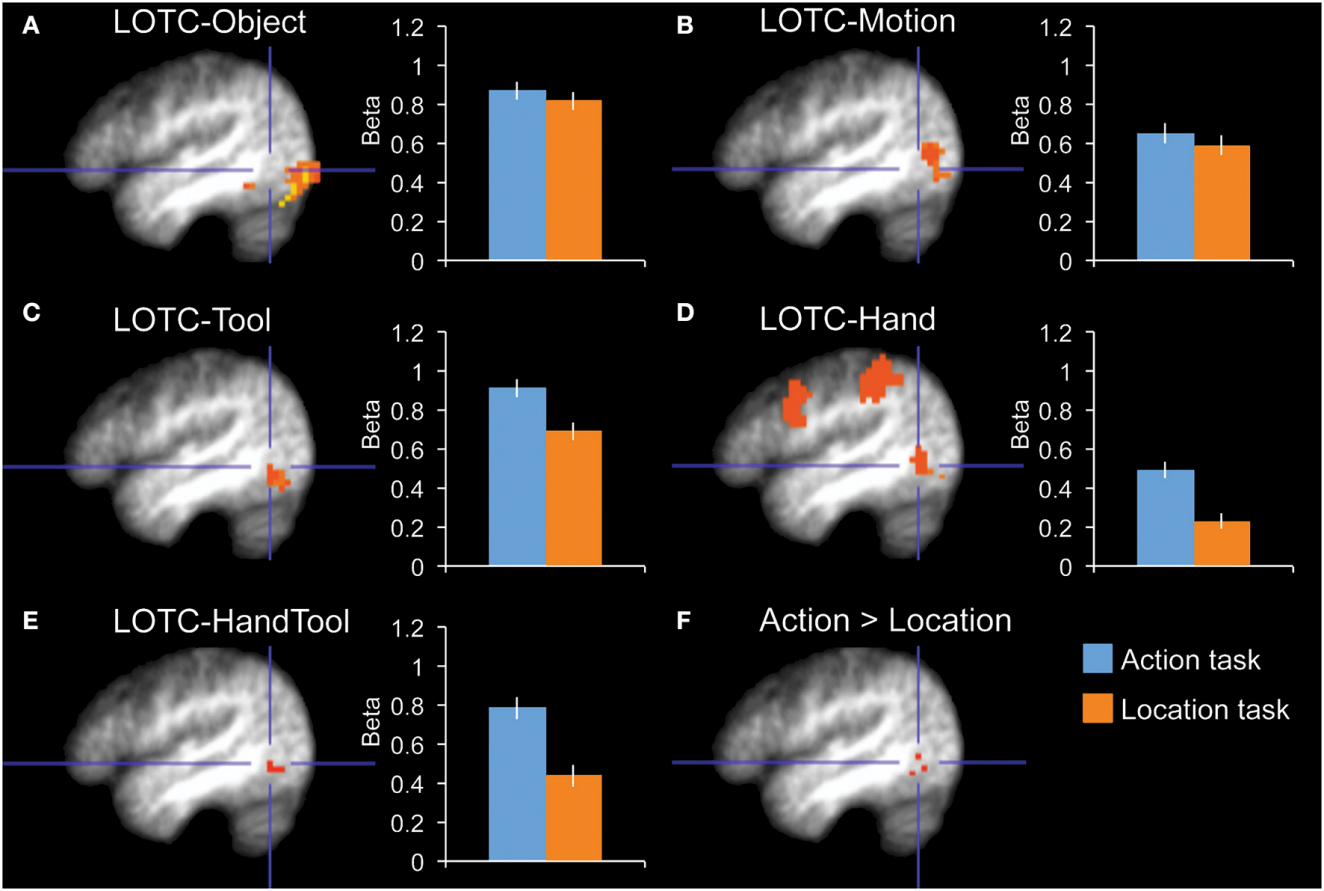
**fMRI results in functionally localized regions in left lateral occipitotemporal cortex**. Bar graphs indicate mean activity (Beta weights) in the displayed ROIs during the action (blue) and location (orange) tasks of the main experiment. **(A)** LOTC-Object (intact > scrambled objects), **(B)** LOTC-Motion (moving > static dots), **(C)** LOTC-Tool (tools > animals), **(D)** LOTC-Hand (hands > animals), **(E)** LOTC-HandTool (tools > animals AND hands > animals), **(F)** the whole-brain group-average contrast action task > location task gave activity in left LOTC, overlapping with tool and hand-selective ROIs. All brain activity maps are shown at *P* < 0.005, displayed on the group-average anatomical scan, at *x* = −46. Blue crosshairs centered on action > location contrast **(F)** are added for spatial reference across panels. Error bars reflect within-subject s.e.m.

**Figure 3:**
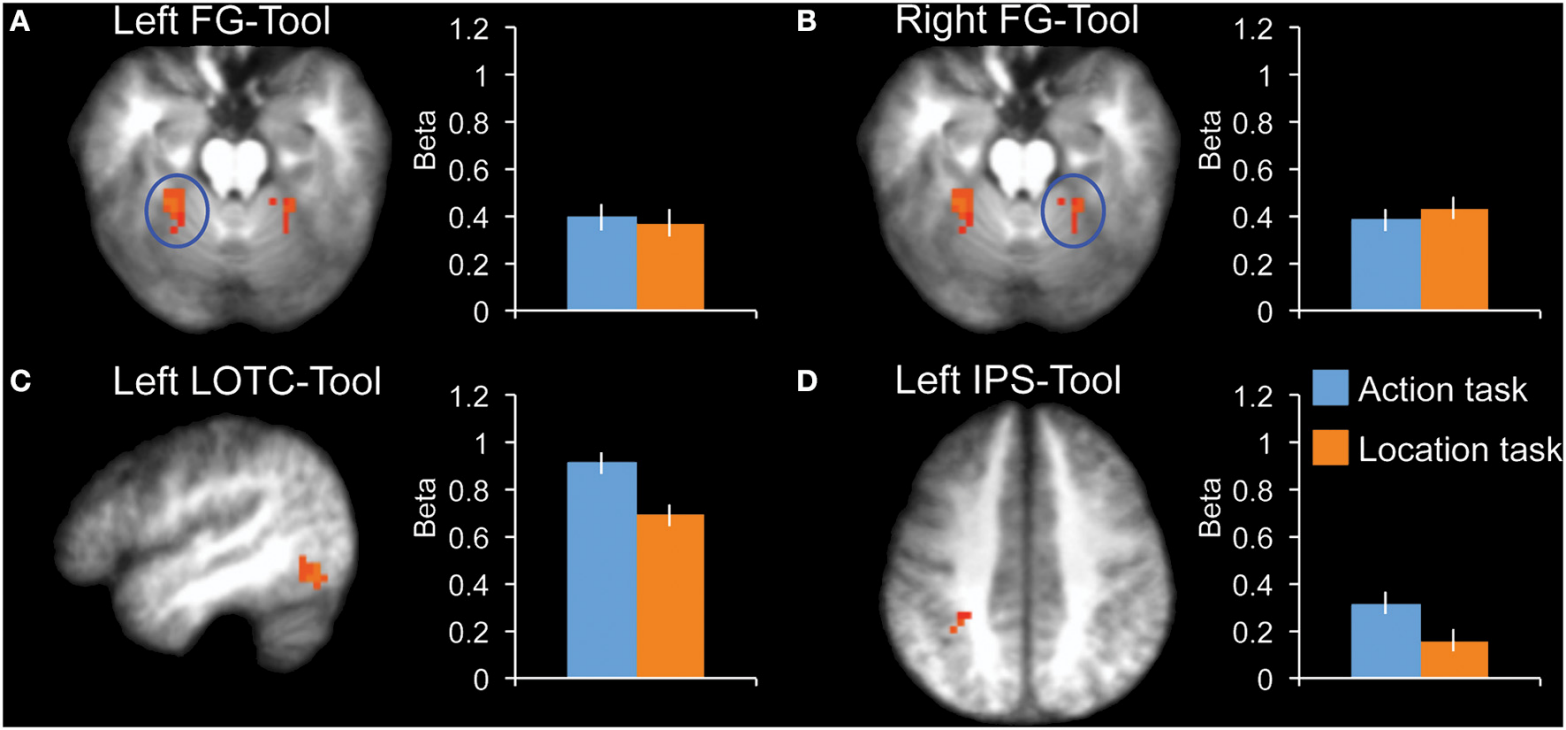
**fMRI results in tool-selective regions, localized by contrasting tools with animals**. Bar graphs indicate mean activity (Beta weights) in the displayed ROIs during the action (blue) and location (orange) tasks of the main experiment. **(A)** Left FG-Tool (*z* = −18), **(B)** Right FG-Tool (*z* = −18), **(C)** left LOTC-Tool (*x* = −46), **(D)** Left IPS-Tool (*z* = 42). All brain activity maps are shown at *P* < 0.005, displayed on the group-average anatomical scan. Error bars reflect within-subject s.e.m.

**Figure 4:**
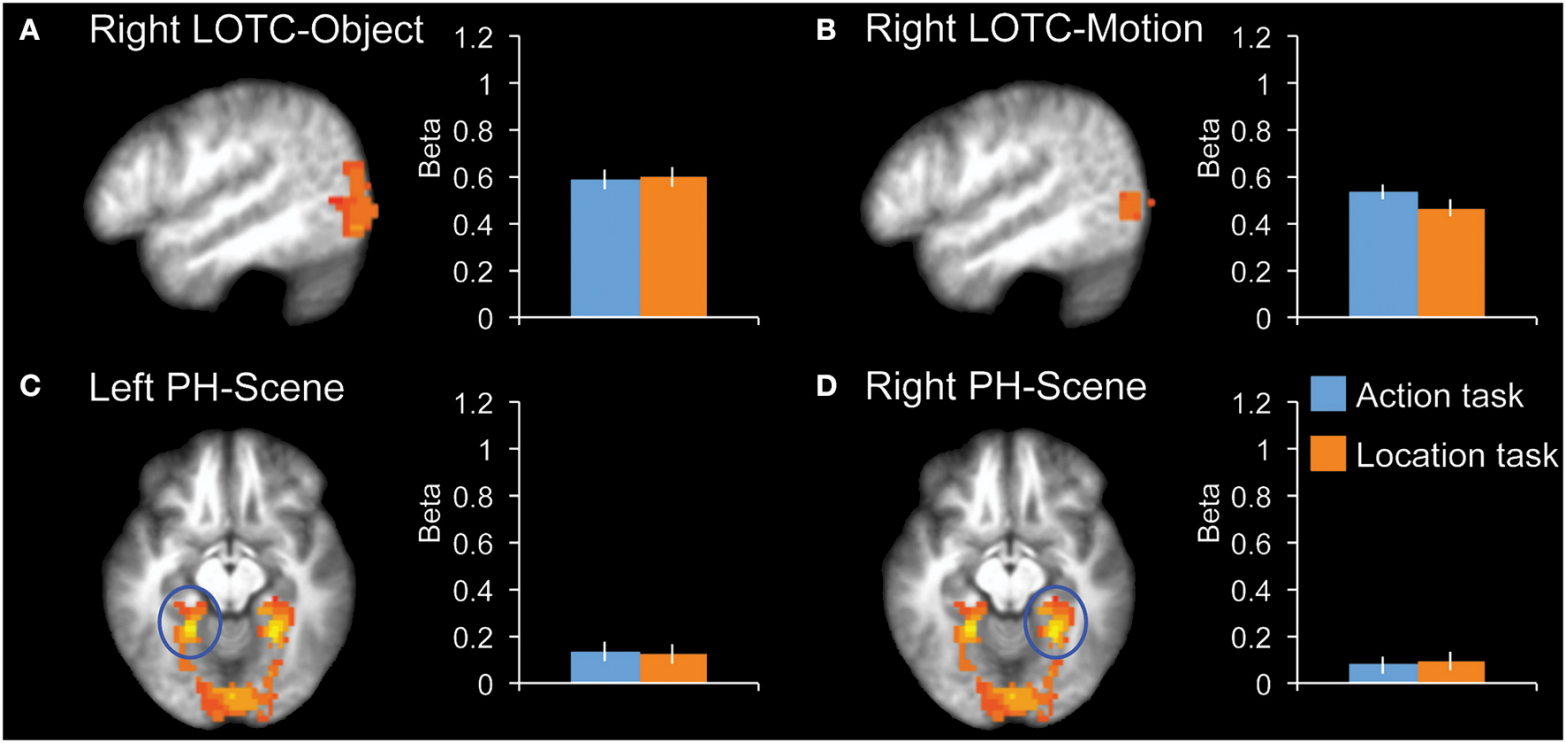
**fMRI results in additional control regions**. Bar graphs indicate mean activity (Beta weights) in the displayed ROIs during the action (blue) and location (orange) tasks of the main experiment. **(A)** Right LOTC-Object (intact > scrambled objects, *x* = −49), **(B)** Right LOTC-Motion (moving dots > static dots, *x* = −49), **(C)** left PH-Scene (scenes > hands + tools + animals, *z* = −9), **(D)** Right PH-Scene (scenes > hands + tools + animals, *z* = −9). All brain activity maps are shown at *P* < 0.005, displayed on the group-average anatomical scan. Error bars reflect within-subject s.e.m.

Regions of interest (ROIs) were defined based on the independent localizer experiments in which 12 of the participants participated. Because not all participants had functional localizer data and because not all ROIs could be defined in those participants who had, ROIs were defined based on group-average data in random-effects analyses (see Supplementary Table 1 for a list of activations from whole-brain analyses of the localizer data). These ROIs were then used to extract data for all participants in the main experiment. All ROIs were defined at a threshold of *P* < 0.005, *t*_(11)_ = 3.5. ROIs were drawn using AFNI software, with individual-subject data extracted from the ROIs using MATLAB. Activity estimates (Beta weights) were averaged across the voxels of an ROI. Statistical analyses were performed in SPSS. We refer to the ROIs by a combination of anatomical and functional characteristics (e.g., LOTC-Tool). The anatomical labels describe the anatomical position of the ROIs (see Figures 2–**4**) rather than a-priori anatomical constraints.

The category localizer served to define our main ROIs. The contrast of tools greater than animals was used to define tool-selective regions in left LOTC (LOTC-Tool; volume = 729 mm^3^), left and right fusiform gyrus (FG-Tool; 1215 and 621 mm^3^), and left intraparietal sulcus (IPS-Tool; 648 mm^3^). The contrast of hands greater than animals was used to define a hand-selective region in left LOTC (LOTC-Hand; 1647 mm^3^). The conjunction of these two contrasts (Bracci et al., 2012) was used to define a region in left LOTC selective for both hands and tools (LOTC-HandTool; 243 mm^3^). The contrast of scenes greater than the average of the other three categories (hands, tools, animals) was used to define the left and right parahippocampal place area (PH-Scene; 5238 and 5589 mm^3^). The OSC localizer (intact objects > scrambled objects) was used to define left and right object-selective regions in LOTC (LOTC-Object; 5427 and 7803 mm^3^). Finally, the MT/MST localizer (moving dots > static dots) was used to define left and right motion-selective regions in LOTC (LOTC-Motion; 2079 and 2673 mm^3^). See earlier work (Downing et al., 2007; Bracci et al., 2012) for detailed analyses of the relation and overlap between hand-, tool-, motion-, and object-selective LOTC regions.

### TMS experiment

#### Participants

Twenty-four healthy adult volunteers (19 females; mean age: 27.7 years; age range: 20–39 years) participated in the TMS experiment. None of these participants had taken part in the fMRI experiment. All participants were right-handed with normal or corrected-to-normal vision, and no history of neurological or psychiatric disease. Fifteen participants were tested at the Center for Mind/Brain Sciences, University of Trento, Italy, while the other nine participants were tested at the School of Psychology, Bangor University, UK. Participants gave written informed consent for participation in the study, which was approved by the human research ethics committees of the University of Trento and Bangor University.

#### Stimuli

The stimuli used in the TMS experiment were the same as those used in the fMRI experiment. Stimuli (280 × 280 pixels, 5°) were presented on a 17-inch LCD monitor (DELL 1908FP-BLK, in Italy), and a Samsung notebook (NP300E5C, in UK) in a dimly lit room. Stimuli were presented using ASF (Schwarzbach, 2011), an add-on to the Psychophysics Toolbox package (Brainard, 1997) in Matlab (Mathworks, Natick, MA, USA). Masks (280 × 280 pixels, 5°) consisted of static noise pattern of black and gray squares (7 × 7 pixels).

#### Task and design of TMS experiment

Participants performed two tasks. In the action task, participants indicated with a button press (using the index and middle finger of the right hand) whether a tool was associated with a rotation or a squeeze movement. In the location task, participants indicated with a button press (using the index and middle finger of the right hand) whether a tool was associated with a garage or a kitchen location. Participants were instructed to respond as fast and accurately as possible.

Participants performed a total of 6 runs; 4 runs with effective TMS and 2 runs with sham TMS. Sham TMS runs were identical to effective TMS runs except that the TMS coil was placed perpendicular to the scalp. For each participant, the order of the 4 effective and 2 sham TMS runs was randomized, with the constraint that the two sham TMS runs were never consecutive.

Each run was divided into two blocks, one block in which participants performed the action task, and one block in which they performed the location task. The order of blocks within runs was alternated across runs such that half the effective TMS runs and half the sham TMS runs started with the action task. In total each participant performed 640 trials. Each trial started with a 1600 ms fixation cross, followed by the picture of a tool presented for 33 ms, which was immediately followed by a mask presented for 600 ms. Participants had to respond as fast as possible, and always within 2000 ms. The next trial started either 2700 or 3000 ms after the offset of the mask (with 50% probability). The brief presentation time of the tool pictures was chosen to make the task sufficiently challenging and to avoid ceiling effects.

#### TMS methods

For correct placement of the TMS coil, structural MRI images (MP-RAGE sequence with 1 × 1 × 1 mm resolution) were acquired for all participants. The position of the TMS coil was co-registered with the participant's reconstructed head, and the location of TMS-stimulation was marked on the reconstructed pial surface of each individual's brain. At the University of Trento we used the BrainVoyager Neuronavigator system (Brain Innovation BV, The Netherlands, version 2.1) combined with a Zebris CMS20S measuring system for real-time motion analysis (Zebris Medical GmbH, Isny, Germany), whereas at Bangor University we used Brainsight (Rogue Research, Montreal, Canada). TMS was applied over hand-/tool-selective left LOTC. For 20 participants, LOTC coordinates were the group-average Talairach coordinates (−46, −68, −2) from a previous study that localized hand-/tool-selective LOTC (Bracci et al., 2012) contrasting hands > chairs and tools > chairs (the mean Talairach coordinates for these contrasts were identical). Talairach coordinates were transformed back into each subject's native space for correct neuronavigation. The other four participants had previously participated in an unrelated fMRI experiment that included a functional localizer of hand-/tool-selective LOTC. For these participants, LOTC was functionally localized with an fMRI localizer experiment in which pictures of tools, hands, and chairs were presented (Bracci et al., 2012). Participants performed two runs, each containing six 14-s blocks per category. Left LOTC was localized by the conjunction of the contrast hands > chairs and the contrast tools > chairs. The mean Talairach coordinates for these participants were: −44, −64, 1.

At the University of Trento, biphasic TMS pulses were applied with a 75-mm figure-of eight coil (MC-B65) and a MagPro × 100 stimulator (MagVenture A/S, Denmark). At Bangor University, biphasic TMS pulses were applied with a 70-mm figure-of eight coil and a Magstim Super Rapid stimulator (Magstim, Whitland, UK). The stimulation intensity during the experiment was set at 120% of the individual resting motor threshold, measured as the intensity that elicited five visible hand movements out of 10 stimulations. This resulted in a TMS intensity that ranged between 42% and 68% of the maximum stimulator intensity.

In the absence of strong timing predictions, we chose a broad window of stimulation, with 3 stimulation times relative to picture onset (30/130 ms, 150/250 ms, and 270/370 ms). These timings were chosen to avoid missing the critical window during which LOTC might be involved in tool processing. For the same reason, on each trial, two TMS pulses were applied with an interval of 100 ms (Rice et al., 2006; Mullin and Steeves, 2011; van Koningsbruggen et al., 2013). Thus, pulses were delivered, on different trials, at three different timings relative to stimulus onset: 30/130 ms, 150/250 ms, and 270/370 ms. These three timings were used for both effective and sham TMS runs. The three TMS timings were each used on 1/3 of the trials, in random order. TMS timing was randomly paired with specific tool pictures. The effects of TMS were found not to significantly depend on the timing of stimulation (*P* > 0.35, for all interactions involving timing as factor). As this might reflect a lack of power to detect such differences, we do not conclude from this null result that each time window is equally important for tool processing. In the absence of significant time differences, we collapsed the data across the different timings. RTs for incorrect trials and RTs that were 2 standard deviations away from a participant's mean RT were excluded from the analyses.

## Results

### fMRI results

A total of 12 ROIs were defined based on three functional localizer experiments (*Materials and Methods*). We tested whether activity in these ROIs differentiated between the action and location tasks.

Our main interest was in the left LOTC. To test whether task-related modulations were specific to hand-/tool-selective regions, we defined four nearby ROIs in the left LOTC: left LOTC-Hand, left LOTC-Tool, left LOTC-Object, and left LOTC-Motion (Figures 2A–D). A 2 × 4 ANOVA with Task and ROI as factors showed a main effect of ROI [*F*_(3, 10)_ = 16.7, *P* < 0.001] and no main effect of Task [*F*_(1, 12)_ = 2.9, *P* = 0.12]. Importantly, there was a significant interaction between Task and ROI [*F*_(3, 10)_ = 5.6, *P* = 0.016], indicating that task-related modulations differed for the ROIs. Follow-up tests in each of the 4 ROIs showed a significantly stronger response during the action task than the location task in LOTC-Hand [*t*_(12)_ = 3.0, *P* = 0.011, Cohen's *d* = 0.83] and LOTC-Tool [*t*_(12)_ = 2.3, *P* = 0.038, Cohen's *d* = 0.65], but not in LOTC-Object [*t*_(12)_ = 0.6, *P* = 0.57, Cohen's *d* = 0.16] or LOTC-Motion [*t*_(12)_ = 0.6, *P* = 0.58, Cohen's *d* = 0.16].

To test whether results differed in nearby hand- and tool-selective LOTC regions, we compared task-related modulation in left LOTC-Hand, left LOTC-Tool, and left LOTC-HandTool (Figure 2E), a region defined as the overlap between LOTC-Hand and LOTC-Tool (*Materials and Methods*). A 2 × 3 ANOVA with Task and ROI as factors showed no interaction between Task and ROI [*F*_(2, 11)_ = 2.0, *P* = 0.18], indicating similar task-related modulation in hand- and tool-selective LOTC regions. The main effect of Task was significant, with stronger responses during the action task than the location task [*F*_(1, 12)_ = 9.1, *P* = 0.011]. There was also a significant main effect of ROI [*F*_(2, 11)_ = 6.7, *P* = 0.013].

To compare effects among tool-selective ROIs, we localized 3 tool-selective ROIs in addition to left LOTC-Tool: left FG-Tool, right FG-Tool, and left IPS-Tool (Figure 3). A 2 × 4 ANOVA with Task and ROI as factors showed a main effect of ROI [*F*_(3, 10)_ = 8.6, *P* = 0.004] and no main effect of Task [*F*_(1, 12)_ = 1.0, *P* = 0.34]. Importantly, there was a significant interaction [*F*_(3, 10)_ = 8.4, *P* = 0.004], indicating that the tasks modulated the ROIs to a different extent. Follow-up tests in each ROI showed a significantly stronger response during the action task than the location task in LOTC-Tool [*t*_(12)_ = 2.3, *P* = 0.038, Cohen's *d* = 0.65], but not in left FG-Tool [*t*_(12)_ = 0.2, *P* = 0.82, Cohen's *d* = 0.06] or right FG-Tool [*t*_(12)_ = −0.5, *P* = 0.63, Cohen's *d* = −0.14]. The left IPS-Tool showed stronger activity during the action task than the location task (Figure 3D), but this difference did not reach significance [*t*_(12)_ = 1.7, *P* = 0.12, Cohen's *d* = 0.46].

We tested for task effects in four additional control regions: right LOTC-Object, right LOTC-Motion, left PH-Scene, and right PH-Scene (Figure 4). None of these regions showed a significant difference between the two tasks (|*t*_12_|< 1.0, *P* > 0.34, for all ROIs).

Finally, an exploratory whole-brain random-effects group analysis (at *P* < 0.005, uncorrected) yielded one cluster for the contrast action task > location task, at the location of hand- and tool-selective LOTC (Figure 2F).

#### TMS results

TMS was applied over left hand-/tool-selective LOTC (*Materials and Methods*), the region that was most strongly modulated by the action task in the fMRI experiment.

**Figure 5:**
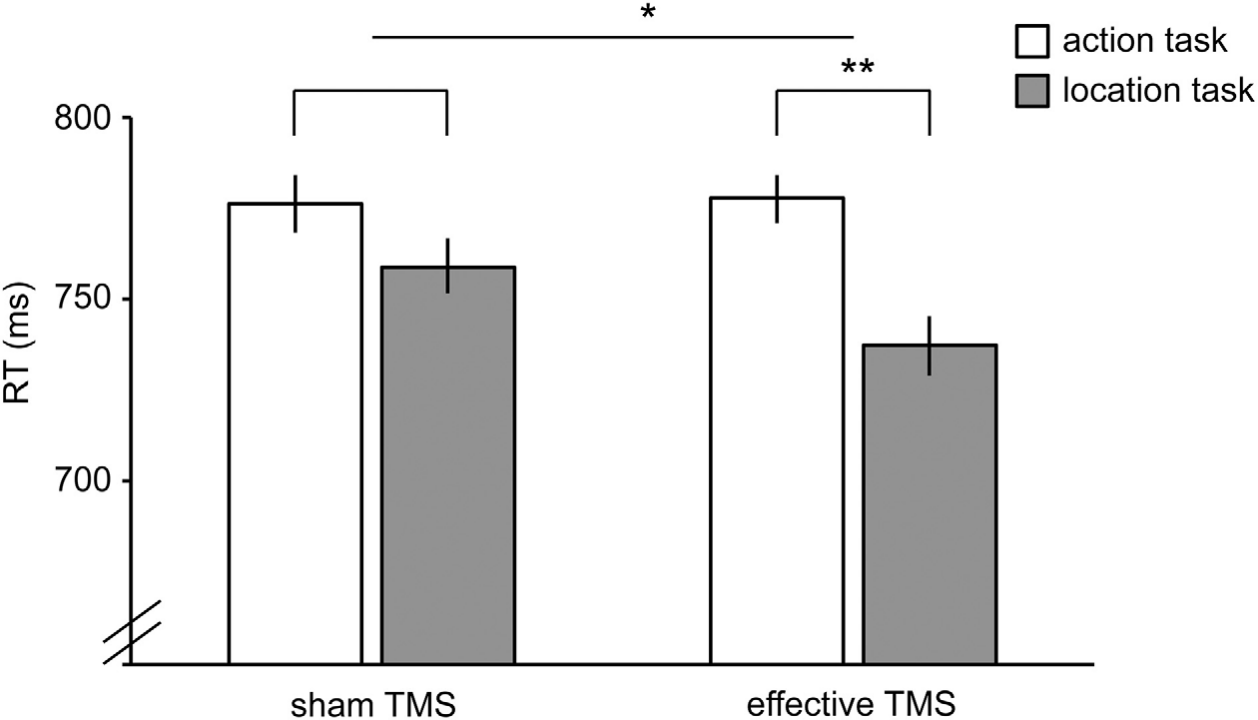
**Results of TMS experiment**. Reaction times in the action task (white bars) and location task (gray bars) during sham TMS (bars on the left) and effective TMS (bars on the right) applied over hand-/tool-selective left LOTC. TMS differentially affected the two tasks, as indicated by a significant TMS x Task interaction. Error bars reflect within-subject s.e.m. ^*^P < 0.05; ^**^P < 0.005.

Reaction time (RT) was analyzed in a 2 × 2 repeated-measures ANOVA, with TMS (sham TMS, effective TMS) and Task (action, location) as factors. There was a significant interaction between TMS and Task [*F*_(1,23)_ = 4.4, *P* = 0.047], indicating that effective TMS (relative to sham TMS) differentially affected performance in the two tasks (Figure 5). Follow-up paired samples *t*-tests showed that effective TMS slowed responses on the action task relative to the location task [*t*_(23)_ = 3.3, *P* = 0.003, Cohen's *d* = 0.67], with no such difference during sham stimulation [*t*_(23)_ = 1.3, *P* = 0.20, Cohen's *d* = 0.27]. For one participant, the effect of TMS on RT was more than 3 standard deviations away from the mean. Without this outlier, the interaction between TMS and Task was highly significant [*F*_(1,22)_ = 11.7, *P* = 0.002]; effective TMS slowed responses on the action task relative to the location task [*t*_(22)_ = 3.2, *P* = 0.004, Cohen's *d* = 0.65], with no such difference during sham stimulation [*t*_(22)_ = 0.9, *P* = 0.38, Cohen's *d* = 0.18].

No significant TMS x Task interaction was found for accuracy [*F*_(1,23)_ = 0.4, *P* = 0.54]. There was no significant main effect of Task [*F*_(1,23)_ = 0.8, *P* = 0.38] and a trend toward a significant main effect of TMS [*F*_(1,23)_ = 3.4, *P* = 0.077], reflecting slightly better performance for sham TMS than effective TMS (Table 1). Results did not change substantially when excluding the outlier.

**Table 1:** **Accuracy and RT for the action task and the location task, during sham TMS and effective TMS**.

	**Action task**	**Location task**	**Action task**	**Location task**
Accuracy, %	90.8 (0.8)	91.6 (0.7)	89.2 (0.8)	90.7 (0.8)
RT, ms	776 (7.9)	759 (7.6)	777 (6.6)	737 (8.3)

*Within-subject s.e.m. are given between brackets*.

## Discussion

Using fMRI and TMS, we tested whether hand-/tool-selective regions in left LOTC are modulated by the task performed on pictures of tools. Results from the fMRI experiment showed that functionally defined tool- and hand-selective LOTC regions, which partly overlapped (Bracci et al., 2012), were more active when participants discriminated the action associated with a tool than when they discriminated the location associated with a tool, providing evidence that left LOTC is involved in processing action-related tool properties. Because the tool pictures presented in both tasks were identical, these results indicate that tool selectivity in LOTC is unlikely to be fully explained by shape differences between tools and other objects (e.g., elongated tool shape; Sakuraba et al., 2012) or by the small size of tools relative to typically used control categories (Konkle and Oliva, 2012). Nearby object- and motion-selective LOTC regions, although strongly responsive to the tool pictures, showed no difference between the two tasks, indicating that the task effects in tool- and hand-selective regions did not reflect general object processing differences between the tasks. Our findings, based on a task manipulation, are in line with a previous fMRI study that used a training manipulation to show increased activity in left LOTC (together with left premotor cortex and left parietal cortex) to pictures of objects that participants had learned to use as tools (Weisberg et al., 2007).

The current fMRI results were confirmed by a TMS study, which showed that effective TMS (relative to sham TMS) differentially affected the action and location tasks performed on the same stimuli, as indicated by a significant interaction between TMS and task. The significant interaction between TMS and task parallels the fMRI finding of stronger LOTC activity for the action task than the location task. It should be noted, however, that there was no significant difference between effective TMS and sham TMS for the action task analyzed separately (Figure 5). The absence of a significant difference between effective TMS and sham TMS on action task performance may reflect known facilitatory effects of effective TMS on response time, such as a general speeding up of responses due to arousal and/or intersensory facilitation (Terao et al., 1997). In our study, such non-specific facilitatory effects may have decreased response times in the effective TMS conditions of both tasks, thereby masking the disruptive effects of TMS on the action task. For this reason, we were primarily interested in the comparison of TMS effects in the action task with TMS effects in the location task (i.e., the interaction between TMS and task); non-specific facilitatory effects of TMS should have equally affected response speed on the two tasks. To provide conclusive evidence for a causal role of the left LOTC in tool action discrimination, however, would require further evidence. Specifically, future studies should test whether tool action judgments are more strongly impaired after effective TMS over left LOTC relative to effective TMS over a nearby control region, thus controlling in another way for non-specific facilitatory effects of TMS.

How might left LOTC contribute to tool action discrimination? The close overlap between hand and tool responses in left LOTC (Bracci et al., 2012) raises the interesting possibility that the conceptual representation of tools may partly consist of the associated hand action representation. That is, discriminating the action of a tool (e.g., knowing that a screwdriver involves a hand rotation) may involve access to the tool-associated hand representation, which may include the mental imagery of tool-associated hand postures. Previous TMS studies have shown that TMS over the extrastriate body area (EBA; Downing et al., 2001), located about 0.5 cm posterior to hand-selective LOTC (Bracci et al., 2010, 2012), selectively impairs detection (van Koningsbruggen et al., 2013) and discrimination of bodies (Pitcher et al., 2009) and body parts, including hands (Urgesi et al., 2004, 2007). It would be interesting for future studies to test, using fMRI and TMS, whether hand discrimination and tool action discrimination reflect the same underlying process in left LOTC, or whether these can be dissociated.

Our fMRI study focused on the hand-/tool-selective LOTC, functionally defined by contrasting activity to pictures of hands and/or tools with animals or chairs (both control categories give a similar localization of this region; Bracci et al., 2012), while participants performed a 1-back repetition detection task. Previous studies that defined tool-selective LOTC (e.g., Chao et al., 1999) have sometimes located this region in the posterior middle temporal gyrus (pMTG). However, in our experience it is typically located inferior/posterior to the MTG, often in the inferior temporal sulcus. It is possible that the location depends on the particular task used for localization, with more semantic tasks (e.g., learning facts about tools; Simmons et al., 2010; Simmons and Martin, 2012) shifting activity superiorly and anteriorly. Indeed, the MTG has been implicated in a variety of semantic tasks, including semantic control and conceptual processing (Whitney et al., 2011; Wei et al., 2012), verb processing (Perani et al., 1999; Shapiro et al., 2006; Willms et al., 2011; Peelen et al., 2012b), action knowledge (Martin et al., 1995; Kable et al., 2005), and access to functional object properties (Bach et al., 2010). It is presently unclear how these regions correspond to the hand-/tool-selective region investigated here. Recent studies have started to dissociate nearby verb-selective, action-selective, body-selective, motion-selective, and object-selective regions in posterior temporal cortex (Downing et al., 2007; Bedny et al., 2008; Valyear and Culham, 2010; Peelen et al., 2012b), but further research is necessary to investigate how hand-/tool-selective LOTC relates to more anterior parts of MTG implicated in other studies.

In the present fMRI study, tool-selective left IPS showed higher activity during the action task than the location task (Figure 3D), but this difference did not reach significance. Interestingly, as described in the Introduction, a previous fMRI study reported significant modulation in left dorsal premotor cortex and left IPS, but not in left LOTC, when contrasting action knowledge with function knowledge of tools (Canessa et al., 2008). There are several differences between our study and the Canessa et al. study. In the action knowledge task of Canessa et al., participants were asked whether two objects (presented simultaneously for 4 s) were used with the same manipulation pattern (e.g., vacuum cleaner and metal detector), while in the present study participants made a hand rotate vs. hand squeeze discrimination on a single object presented for 1.2 s. The strong focus on the specific hand action associated with a tool in our study (rotate vs. squeeze) may have amplified responses in hand-/tool-selective left LOTC. An additional difference between our study and the Canessa et al. study is that in the present study tool action processing was contrasted with tool location processing, while in the Cannessa et al. study tool manipulation knowledge was contrasted with tool function knowledge. It is plausible that LOTC is also activated when processing function properties of tools, which would account for the weak activity for the contrast between tool function and tool manipulation tasks. Indeed, other imaging studies that contrasted manipulation with function knowledge of manipulable objects similarly found increased activity in parietal cortex but not in LOTC (Kellenbach et al., 2003; Boronat et al., 2005; Bach et al., 2010).

An interesting open question concerns the functional interactions between tool-selective regions in LOTC and parietal cortex. These regions are functionally connected (Bracci et al., 2012; Simmons and Martin, 2012) and also share functional characteristics; for example both regions respond selectively to static depictions of both body effectors and object effectors (Bracci and Peelen, 2013). Both regions likely also contribute unique aspects to tool perception and tool use. For example, work with neurological patients has provided evidence that regions in the left parietal cortex are critical for processing dynamic and possibly motoric aspects of tool actions (Kalenine et al., 2010; Buxbaum et al., 2014; Vingerhoets, 2014), while the occipitotemporal cortex is critical for the representation of semantic action knowledge (Tranel et al., 2003; Kalenine et al., 2010) and postural components of tool-related actions (Buxbaum et al., 2014), in line with the current results. Future studies could use TMS to test the causal involvement of LOTC and parietal cortex in the discrimination of both dynamic and static tool actions.

In a recent study (Peelen and Caramazza, 2012a), we found that multivoxel activity patterns in the anterior temporal lobes (ATLs) carry information about both object-associated location (kitchen vs. garage) and object-associated action (rotate vs. squeeze). For example, activity patterns were relatively similar for objects that are both associated with a rotation action (e.g., a corkscrew and a screwdriver). Because information was computed at the level of action category (rotate vs. squeeze), these results likely reflected categorical representations that generalize across specific visuomotor features. For example, while both a screwdriver and a corkscrew involve a wrist rotation movement, their specific motor patterns and hand postures—determined by the specific visual form of the objects in question—are quite distinct. The present study, showing that the left LOTC is involved in tool action discrimination, complements these results: rather than housing generalized category-level representations of tool actions, LOTC may represent more specific visuomotor representations associated with individual tools. Access to such representations is required for performing higher-order categorization, and thus for performing the current action task. More generally, the two sets of results suggest that the functional roles of LOTC and ATL are related hierarchically, with LOTC reflecting an earlier, less abstract and less general level of representation than the ATL.

It remains to be fully determined what exactly LOTC represents about tools. One possibility is that LOTC represents the hand/arm motion patterns associated with tools (Beauchamp et al., 2002; Weisberg et al., 2007; Orlov et al., 2014), perhaps particularly for tools that extend the hands' reach (Bracci and Peelen, 2013). Alternatively, LOTC may comprise static representations, or “snapshots,” of hand/arm postures associated with tools (Vangeneugden et al., 2014) or representations of hand-appropriate shape/size features of objects (Konkle and Caramazza, 2013). Hand postures and movements are in large part determined by the shape of a tool (e.g., the diameter of a tool's grip). Therefore, these suggestions point to a role for LOTC in linking tool shape and hand/arm representations to support tool action discrimination.

To conclude, the present study provides converging evidence from fMRI and TMS that the left LOTC is more strongly involved in the discrimination of actions than in the discrimination of locations associated with visually presented tools. Furthermore, the finding that left LOTC contributes to the discrimination of tool-associated hand actions suggests that tool selectivity in left LOTC is driven, at least in part, by action-related tool properties.

### Conflict of interest statement

The authors declare that the research was conducted in the absence of any commercial or financial relationships that could be construed as a potential conflict of interest.
