# Supplementary material for: Left occipitotemporal cortex contributes to the discrimination of tool-associated hand actions: fMRI and TMS evidence
Source: Front Hum Neurosci. 2014 Aug 5;8:591. doi: 10.3389/fnhum.2014.00591 (PMC4122187; doi:10.3389/fnhum.2014.00591)
Supplement: Supplementary file 1 [file DataSheet1.DOCX]

***Supplementary Material***

**Left occipitotemporal cortex contributes to the discrimination of tool-associated hand actions: fMRI and TMS evidence**

**Francesca Perini^1^, Alfonso Caramazza^1,2^, Marius V. Peelen^1,^***

^1^Center for Mind/Brain Sciences, University of Trento, 38068 Rovereto (TN), Italy. ^2^Department of Psychology, Harvard University, Cambridge, MA 02138, USA

*** Correspondence:** Marius V. Peelen, Center for Mind/Brain Sciences, University of Trento, Corso Bettini 31, 38068 Rovereto (TN), Italy, phone: +39 0464 808718, e-mail: marius.peelen@unitn.it

**Supplementary Table 1.** List of activated clusters in whole-brain random-effects group analyses of localizer data. Whole-brain activation maps were thresholded at *p* < 0.0005, with a minimum cluster size of 150 mm^3^. Coordinates are of a cluster’s center of mass, in Talairach space.

|  | x | y | z | Volume  (mm^3^) | Mean T |
| --- | --- | --- | --- | --- | --- |
| *Tools > Animals* | |  |  |  |  |
|  | -26.0 | -37.4 | -18.5 | 324 | 5.5 |
|  | -46.5 | -58.1 | -6.1 | 243 | 5.5 |
|  |  |  |  |  |  |
| *Hands > Animals* | |  |  |  |  |
|  | -38.8 | -38.4 | 42.2 | 3294 | 6.2 |
|  | 29.7 | -50.9 | 46.2 | 2997 | 5.6 |
|  | 28.6 | -4.2 | 58.1 | 756 | 5.8 |
|  | -48.1 | 5.6 | 33.9 | 594 | 6.1 |
|  | -51.4 | -56.0 | 1.3 | 351 | 5.3 |
|  | -27.8 | -8.1 | 57.3 | 324 | 6.0 |
|  | 41.6 | 29.1 | 25.9 | 297 | 5.6 |
|  | 50.9 | -56.6 | -7.2 | 243 | 5.2 |
|  | 44.4 | -1.9 | 39.4 | 162 | 5.4 |
|  |  |  |  |  |  |
| *Scenes > [Animals + Tools + Hands]* | | | |  |  |
|  | 1.2 | -86.2 | -1.9 | 5859 | 6.1 |
|  | -16.5 | -47.1 | -0.9 | 5211 | 6.2 |
|  | 23.0 | -41.8 | -5.8 | 4077 | 6.2 |
|  | 21.3 | -71.7 | -8.1 | 1080 | 5.7 |
|  | -24.2 | -89.8 | 13.0 | 432 | 6.0 |
|  | -24.0 | -62.6 | -11.6 | 297 | 5.5 |
|  | 38.9 | -76.8 | 13.9 | 216 | 5.9 |
|  |  |  |  |  |  |
| *Intact > Scrambled* | |  |  |  |  |
|  | -28.1 | -38.0 | -14.8 | 3294 | 6.4 |
|  | -40.2 | -72.6 | -5.0 | 3213 | 5.8 |
|  | 42.7 | -72.8 | -7.9 | 1998 | 6.2 |
|  | 30.3 | -36.4 | -15.2 | 1377 | 5.8 |
|  | 45.9 | -65.9 | 15.1 | 918 | 5.7 |
|  | -26.0 | -4.3 | -9.1 | 675 | 5.8 |
|  | 12.5 | -59.9 | 20.4 | 243 | 5.9 |
|  | 39.4 | -53.4 | -17.1 | 216 | 5.4 |
|  | -14.6 | -55.9 | 12.0 | 162 | 5.6 |
|  |  |  |  |  |  |
| *Motion > Static* | |  |  |  |  |
|  | 24.9 | -85.7 | 14.6 | 756 | 6.0 |
|  | -33.8 | -80.1 | 4.9 | 621 | 5.6 |
|  | 38.2 | -74.3 | 8.5 | 513 | 5.6 |
|  | 12 | -83.2 | -1.6 | 351 | 5.8 |
|  | 46.9 | -66.7 | 2.5 | 351 | 6.4 |
|  | 21.7 | -73.5 | 36.3 | 297 | 6.1 |
|  | 22.7 | -66.3 | -6.9 | 270 | 5.6 |
|  | -9.6 | -87.5 | -3.8 | 270 | 5.5 |
|  | 11.8 | -72.9 | -7.2 | 243 | 6.1 |
|  | -19.1 | -85.6 | 10.6 | 189 | 5.3 |
|  | -22.7 | -83.7 | 17.5 | 189 | 6.1 |
|  | 29.5 | -75.5 | 20 | 162 | 5.2 |
|  | 13.5 | -89.9 | 25.5 | 162 | 5.5 |
